# Synergism, Bifunctionality, and the Evolution of a Gradual Sensory Trade-off in Hummingbird Taste Receptors

**DOI:** 10.1093/molbev/msab367

**Published:** 2022-01-03

**Authors:** Glenn Cockburn, Meng-Ching Ko, Keren R Sadanandan, Eliot T Miller, Tomoya Nakagita, Amanda Monte, Sungbo Cho, Eugeni Roura, Yasuka Toda, Maude W Baldwin

**Affiliations:** 1 Evolution of Sensory Systems Research Group, Max Planck Institute for Ornithology, Seewiesen, Germany; 2 Macaulay Library, Cornell Lab of Ornithology, Ithaca, NY, USA; 3 Department of Agricultural Chemistry, School of Agriculture, Meiji University, Kawasaki, Kanagawa, Japan; 4 Proteo-Science Center, Ehime University, Matsuyama, Ehime, Japan; 5 Department of Behavioral Neurobiology, Max Planck Institute for Ornithology, Seewiesen, Germany; 6 Centre for Nutrition and Food Sciences, Queensland Alliance for Agriculture and Food Innovation, The University of Queensland, St Lucia, Australia

**Keywords:** sensory receptor evolution, functional profiling, sensory trade-off, ancestral reconstruction, hummingbird taste, molecular evolution

## Abstract

Sensory receptor evolution can imply trade-offs between ligands, but the extent to which such trade-offs occur and the underlying processes shaping their evolution is not well understood. For example, hummingbirds have repurposed their ancestral savory receptor (T1R1–T1R3) to detect sugars, but the impact of this sensory shift on amino acid perception is unclear. Here, we use functional and behavioral approaches to show that the hummingbird T1R1–T1R3 acts as a bifunctional receptor responsive to both sugars and amino acids. Our comparative analyses reveal substantial functional diversity across the hummingbird radiation and suggest an evolutionary timeline for T1R1–T1R3 retuning. Finally, we identify a novel form of synergism between sugars and amino acids in vertebrate taste receptors. This work uncovers an unexplored axis of sensory diversity, suggesting new ways in which nectar chemistry and pollinator preferences can coevolve.

## Introduction

Sensory trade-offs can arise when an organism increases specialization in one modality at the expense of another, such as changes in the visual system observed in bats that rely on sophisticated echolocation (De Gutierrez et al. 2018), or the loss of olfactory receptors seen in primates following the acquisition of acute vision (Niimura et al. 2018). Similar trade-offs can also occur within a single sensory modality, for instance when certain stimuli are preferentially detected and sensitivity to other stimuli are reduced. The changes in tuning breadth documented in chemosensory receptors such as taste and olfactory receptors (Baldwin and Ko 2020) may reflect specialization to specific aspects of an organism’s dietary niche, resulting in a reduced ability to detect other sensory cues.

The savory, or umami, receptor of vertebrates is a heterodimer composed of two G-protein coupled receptors, T1R1 and T1R3. In hummingbirds and in songbirds, the T1R1–T1R3 receptor, ancestrally responsive likely only to amino acids (Nelson et al. 2002; Baldwin et al. 2014), has convergently undergone a functional shift, enabling these species to detect sugars (Baldwin et al. 2014; Toda et al. 2021b). A common way that novel functions emerge is through gene duplication and neofunctionalization of one duplicate, which can permit the ancestral function to be retained (Innan and Kondrashov 2010); in avian sugar sensing, however, a single receptor may be responsible for detecting both sugars and amino acids, potentially requiring a trade-off. In songbirds, amino acid responses are robust in some species but reduced in others (Toda et al. 2021b). Since receptors of only a single hummingbird have previously been tested, diversity within hummingbirds is unexplored, and whether hummingbirds can respond to and prefer amino acids is not known. Moreover, it is unclear if the switch between amino acid and sugar preference happened simultaneously, suggesting a functional constraint of the protein, or was selected for gradually over the evolution of the hummingbird lineage.

The immense hummingbird radiation consists of over 300 species and spans a wide geographic and elevational distribution, from Alaska to Patagonia (Rahbek and Graves 2000; Graham et al. 2009). Different species encounter vastly different floral communities (see supplementary table 1, Supplementary Material online), and as plant nectar chemistry varies extensively, coevolutionary relationships between pollinator taste preferences and nectar chemistry are likely (Johnson and Nicolson 2008). Despite relying primarily on nectar sugars, hummingbirds also require amino acids to cover their nitrogen requirements—therefore, insects are an essential part of the hummingbird diet (Stiles 1995; López-Calleja et al. 2003). Many plant nectars also contain amino acids (Baker and Baker 1986): although a few species contain unusually high amino acid levels (Nicolson 2007), nectars of many bird-pollinated plants contain amino acids at low concentrations (Gardener and Gillman 2001; Nicolson 2007), but the functional significance is unclear.

Here, we asked whether the retuning of the hummingbird umami receptor to sense sugars resulted in a trade-off with amino acid sensing. We examined receptor responses to a broad panel of amino acids and sugars from representatives of nearly all major hummingbird clades, as well as from ancestrally reconstructed receptors, to examine response diversity and to investigate the relative timing and molecular basis of functional shifts. We also identify an unexpected type of synergism not known to be exhibited by vertebrate T1Rs. Our results underscore the importance of functionally profiling diverse representatives from a clade to understand the molecular mechanisms and evolutionary timing of sensory changes. In addition, these findings suggest an unexplored role for nectar amino acids and reveal a novel way in which plants may select sets of pollinators, enabling nectar chemistry and pollinator preferences to coevolve.

## Results

### Bifunctional Hummingbird Taste Receptors Retain Amino Acid Sensitivity during Retuning

**Fig. 1.**
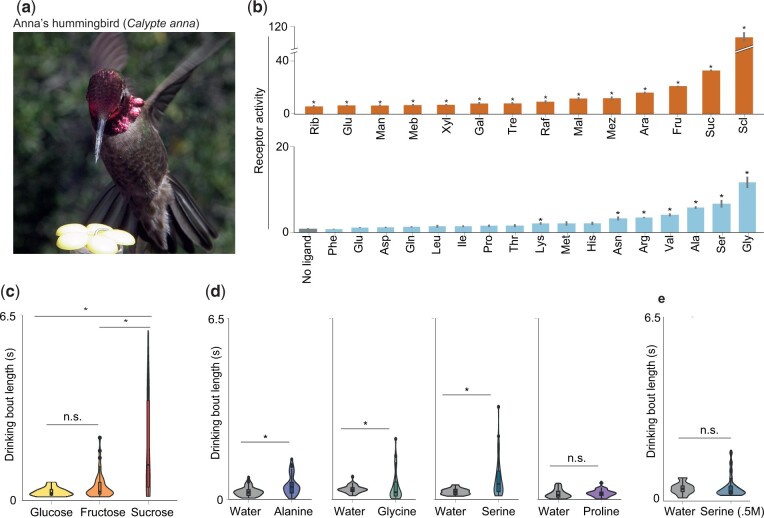
Anna’s hummingbird T1R1–T1R3 is a bifunctional receptor and drives appetitive behavior. (*a*) Receptor responses and behavioral preferences of Anna’s hummingbirds (*Calypte anna*) were assessed (Photo credit: M. Baldwin). (*b*) Responses of T1R1–T1R3 to a panel of sugars and amino acids: sugars (100 mM, *n* = 6) are shown in orange and amino acids (100 and 50 mM; see Materials and Methods for a list of all stimuli) in blue (all ligands: *n* = 6, *P < 0.05). (*c*, *d*) Wild hummingbirds were presented simultaneously with test solutions, and drinking bout length was recorded. (*c*) In tests of equimolar (500 mM) carbohydrate solutions, sucrose was significantly preferred over fructose or glucose (Kolmogorov–Smirnov test; *P* < 0.05). (*d*) Amino acid solutions (1.5 M) containing receptor agonists (alanine, serine, and glycine) but not proline elicited longer bouts compared with paired water controls (Anna’s hummingbird males, Kolmogorov–Smirnov test; *P* < 0.05; see also, supplementary fig. 1 and table 4, Supplementary Material online, for sample sizes). (*e*) Lower concentration (500 mM) solutions of serine evoked some long bouts, but the difference between bout lengths compared with water controls was not significant.

As our previous results suggested that hummingbird taste receptors may retain some weak responses to amino acids (Baldwin et al. 2014), we proceeded to explore the extent to which T1R1–T1R3 tuning toward sugars implied a loss of amino acid sensitivity. We examined the taste receptor responses of Anna’s hummingbird (fig. 1*a*) to an extended panel of amino acids and sugars, and, in addition to strong sugar responses, observed clear responses to a subset of amino acids (fig. 1*b*). Because both ligand types activate the same receptor pair, we predicted that amino acids, like carbohydrates, may also drive appetitive behavior. We examined taste preferences of a population of wild black-chinned and Anna’s hummingbirds (*Archilochus alexandri* and *Calypte anna*) for different sugars and amino acids. In accordance with receptor responses, both species exhibited strong preferences for sucrose compared with fructose and glucose (fig. 1*c* and supplementary fig. 1*a–c*, Supplementary Material online) (although fructose and glucose elicit appetitive behavior when presented at high concentrations (Baldwin et al. 2014), and fructose is preferred when sucrose is absent, supplementary fig. 1*d*, Supplementary Material online). Strikingly, high concentrations (but not low; fig. 1*e*) of three amino acids that activated the receptor most strongly—alanine, serine, and glycine—provoked long drinking bouts; this behavior resembled that elicited by carbohydrates (fig. 1*d* and supplementary fig. 1*e*, Supplementary Material online). By contrast, proline, an amino acid that is not a receptor agonist, was not preferred over water controls (fig. 1*d* and supplementary fig. 1*e*, Supplementary Material online). These responses are consistent with predictions from studies of taste coding: the activation of the taste cell is of primary importance for determining taste qualities, and information from taste cells is generally transmitted to the central nervous system in a labeled-line fashion (Zhao et al. 2003). Different bitter taste receptors respond to diverse chemicals, but because these receptors are coexpressed in the same taste cell, all bitter agonists evoke a single bitter percept (Mueller et al. 2005). Similarly, if distinct chemicals activate the same taste receptor pair—and therefore activate the same taste cell—then they may be indistinguishable (Zhao et al. 2003; Mueller et al. 2005). Unlike humans, hummingbirds may therefore perceive amino acids and sugars as a single, appetitive taste.

### Functional Diversity across the Hummingbird Radiation and the Evolution of a Sensory Trade-Off

**Fig. 2.**
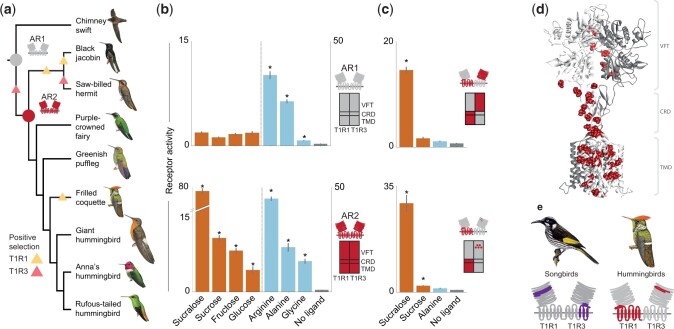
Functional testing of ancestral taste receptors reveals acquisition of sugar responses early in the hummingbird radiation. (*a*) Cladogram showing the relationships between all Apodiformes (hummingbirds and swifts) tested in this study (following McGuire et al. [2014]). Species were chosen to represent eight of the nine major hummingbird clades, including the topazes (black jacobin), the hermits (saw-billed hermit), mangos (purple-crowned fairy), brilliants (greenish puffleg), coquettes (frilled coquette), the monotypic giant hummingbird, the bees (Anna’s hummingbird), and the emeralds (rufous-tailed hummingbird). The reconstructed ARs of the common ancestor of the swift and hummingbirds are shown in gray (AR1), and the ancestral hummingbird receptor is in red (AR2). Triangles indicate branches with evidence for positive selection. (*b*) Responses of ARs to sugars and sweeteners (sucralose, sucrose, fructose, and glucose; orange) and amino acids (alanine, glycine, arginine; blue). AR1 responds only to amino acids, whereas AR2 responds robustly both to sugars and sweeteners as well as amino acids (*n* = 6, mean±SE; *P < 0.05 one-tailed *t-*test). Schematics representing the three main receptor domains are next to each panel: VFT, Venus flytrap domain; CRD, cysteine-rich domain, TMD, transmembrane domain. (*c*) Receptor chimeras between AR1 and AR2 demonstrate an important role of both T1R1 CRD-TMD, as well as five residues in T1R3 VFT. (*d*) Homology model of AR2, depicting differences between ancestors shown in (*c*). Dark red: 40 residues differing between AR1 and AR2 in T1R1 CRD-TMD; light red: five residues in the ligand-binding region of T1R3. (*e*) Similar domains—both VFT and CRD-TMD—but from different members of the heterodimer are involved in the detection of sugars and sweeteners in songbirds (represented by a New Holland honeyeater) and hummingbirds (represented by a frilled coquette), providing a clear example of convergence on the level of the tertiary structure. Illustrations of birds in panels (*a*) and (*e*) reproduced with permission of Lynx Edicions.

To investigate both the timing of the initial shift to sugar sensing as well as the diversity of taste responses across different hummingbird clades, we amplified receptor sequences from eight representatives of major hummingbird clades and reconstructed ancestral receptors (ARs) at two points in the phylogeny (fig. 2*a* and supplementary fig. 2, Supplementary Material online). Functional profiling revealed that the ancestral taste receptor of all hummingbirds (AR pair 2, or AR2), but not the ancestral taste receptor of the hummingbird-swift common ancestor (AR1), responded strongly to sugars (fig. 2*b*), indicating that the acquisition of the carbohydrate response occurred at least 22 Ma, at the base of the hummingbird radiation after the divergence from the common ancestor with swifts, which occurred much earlier (around at least 42 Ma) (McGuire et al. 2014).

As the two ancestral pairs contained the critical residues responsible for the gain in sugar sensing, we employed a chimeric dissection strategy coupled with homology modeling to confirm a key role for residues near the principal ligand-binding region, the extracellular Venus-fly trap (VFT) domain of T1R3 (Baldwin et al. 2014) (fig. 2*c* and supplementary fig. 2*c–i*, Supplementary Material online). Consistent with results in our earlier study suggesting that T1R1 also contributed to sugar sensing, we identified a clear role for the cysteine-rich (CRD) and transmembrane (TMD) domain of the other heterodimeric partner, T1R1 (fig. 2*c* and supplementary fig. 2*c–i* and supplementary table 3, Supplementary Material online) in the response to sucralose. This combination of critical domains represents an interesting example of convergence with the independent acquisition of sweet taste seen in the songbird radiation: in addition to the modifications we have described to distinct ligand-binding domains (T1R3 in hummingbirds and T1R1 in songbirds), we now show that the CRD-TMD region of the opposite paralog (T1R1 in hummingbirds; fig. 2*c–d*, T1R3 in songbirds; Toda et al. 2021b) also plays a critical role in both radiations. Each acquisition of carbohydrate and sweetener detection therefore requires modifying a combination of both domains, but hummingbirds and songbirds recruit alternate paralogs in a mirror-image fashion (fig. 2*e*).

**Fig. 3.**
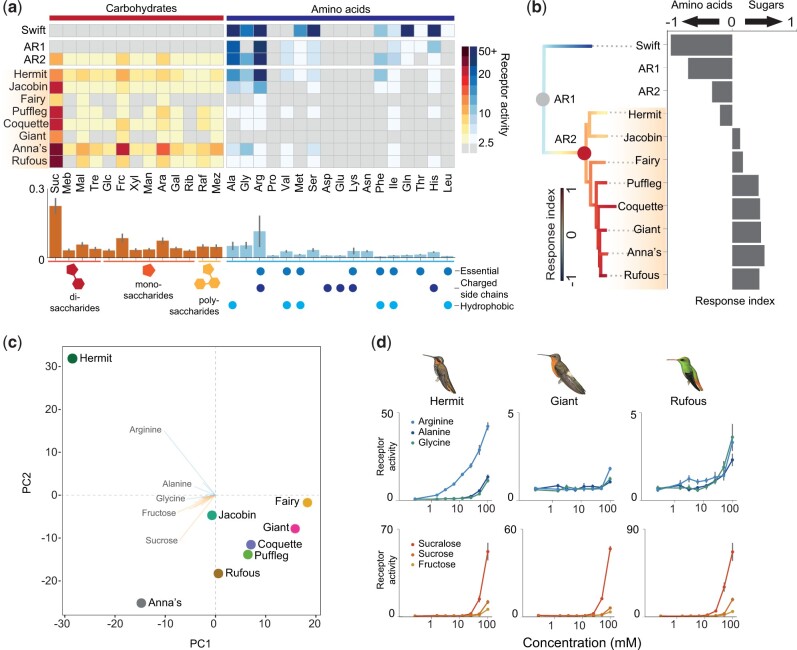
Functional diversity across the hummingbird radiation and the evolution of a sensory trade-off. (*a*) Responses of apodiform T1Rs to a panel of sugars and amino acids. Sugars (100 mM, *n* = 6) are shown in orange and amino acids (100 and 50 mM; see Materials and Methods) in blue (all ligands: *n* = 6). The normalized grand mean (divided by sucralose response) of the response across extant hummingbirds for each ligand is shown (see supplementary fig. 4*c*, Supplementary Material online, for nonnormalized responses). Sucrose elicits the strongest response of all tested carbohydrates; amino acid responses vary across species, with the strongest responses seen to arginine, alanine, and glycine (see supplementary fig. 4 and supplementary table 2, Supplementary Material online). Sugars are classified as di-, mono-, and polysaccharides; amino acids are categorized according to polarity or hydrophobicity, or whether or not they are able to be synthesized endogenously (nonessential or essential data for birds taken from Klasing [1998])—no clear associations are seen between receptor responses and properties of tested ligands. (*b*) An index of the relative average sugar-to-amino acid response (see Materials and Methods) across the tested species (including ancestors) demonstrates a functional shift from responding more strongly to amino acids in the swift and ancestors to responding more strongly to carbohydrates in derived hummingbirds: ancestral reconstruction of this index is depicted on the cladogram (left); index values are shown on the right. (*c*) Phylogenetic principal components analysis (phyloPCA) excluding the swift: the hermit (and to a lesser extent, the jacobin) is distinct from the other tested species. Amino acid eigenvectors are colored blue, eigenvectors for sugars are colored orange. (*d*) Dose–response curves for three species (saw-billed hermit, giant hummingbird, and rufous-tailed hummingbird, *n* = 6 per concentration, mean±SE) display dramatic differences in the magnitudes of amino acid responses across hummingbirds, compared with relatively constant responses to carbohydrates.

In addition to identifying a new critical domain in the acquisition of hummingbird sugar detection, AR profiling also suggested a prominent role for amino acid responses in early hummingbirds. Interestingly, AR2 receptors also displayed strong responses to amino acids, far higher than those observed in Anna’s hummingbirds; average amino acid responses were even slightly elevated compared with AR1. To explore the amino acid response across hummingbirds in more detail, as well as to test whether responses to major nectar sugars varied across the radiation we next examined the functional responses of eight hummingbird species, as well as the receptors of a swift (the closest relatives to hummingbirds) (fig. 3*a*). Representing eight of the nine major clades of hummingbirds (McGuire et al. 2014), these species differ in body mass, as well as in elevation and geographic distribution (supplementary table 1, Supplementary Material online).

Substantial diversity in responses to carbohydrates and amino acids was observed across the eight hummingbirds. Strikingly, taste receptors of all tested species responded most prominently to sucrose (fig. 3*a*), and responses to other carbohydrates varied: arabinose, for instance, evoked stronger responses from Anna’s hummingbird receptors compared with other species (fig. 3*a* and supplementary table 2, Supplementary Material online). Amino acid responses also exhibited substantial variation. The chimney swift receptor was broadly tuned, like receptors from rodents and some primates, but unlike the narrowly tuned receptor of humans (Toda et al. 2013; Toda et al. 2021a). Swift receptors responded strongly to certain amino acids that failed to activate hummingbird receptors, such as glutamine (fig. 3*a*); conversely, with the exception of valine, hummingbird receptors responded to subsets of amino acids that activated the swift’s T1Rs. Within hummingbirds, amino acid responses varied dramatically both in magnitude as well as in tuning breadth, with most species retaining clear responses to multiple amino acids, underscoring the bifunctionality of hummingbird T1R1–T1R3.

The strongest amino acid responses were seen in the hermit and the jacobin, two members of groups that are sister to the rest of hummingbirds. In addition to a robust sugar response, the saw-billed hermit in particular exhibited high amino acid responses, which, importantly, were similar in magnitude to responses of both ARs (fig. 3*a* and supplementary fig. 4*a*, Supplementary Material online). Although our limited taxon sampling restricts formal testing of functional shifts (see Materials and Methods), changes in a response index that captures the relative amino acid to sugar responses suggest that with the increase in relative sugar responses, the robust (and swift-like) amino acid response seen most markedly in the hermit appears to be lost after divergence from the rest of the radiation (fig. 3*b*). The hermit in particular also appears distinct from other hummingbirds by phylogenetic principal component analysis (phyloPCA; Revell 2012; fig. 3*c*), highlighted by dose–response curves underscoring the variation in amino acid sensitivity (fig. 3*d*). Although all receptors are activated by sucralose and sucrose (enabling a comparison of normalized responses to control for differences in transfection efficiency, see fig. 3*a* and supplementary fig. 4*a* and supplementary table 2, Supplementary Material online), especially strong amino acid responses are seen in the hermit; in contrast, amino acid responses of the giant hummingbird are especially reduced.

Our survey of published diet information as well as image databases revealed a diversity of host plant families visited by the hummingbirds we tested (supplementary table 1, Supplementary Material online). Sucrose, fructose, and glucose are the major components of most plant nectars, and although the majority of hummingbird-pollinated flowers have sucrose-dominant nectar (Nicolson and Fleming 2003), much variation exists (even within plants of the same family; Nicolson et al. 2007), and nectars high in mixtures of fructose and glucose are not uncommon. In the few studies where a broader panel of sugars have also been examined, additional carbohydrates (besides sucrose, glucose, and fructose) have also been documented in many nectars, which together with amino acids, may represent important nectar components (Baker and Baker 1973; Gardener and Gillman 2002; Nicolson et al. 2007). Differences in nectar composition may therefore shape taste receptor responses.

### Unexpected Synergism between Amino Acids and Sugars

Whether hummingbird amino acid responses are simply a relic from the ancestral response profile of the T1R1–T1R3 receptor inherited from the common ancestor of hummingbirds and swifts, or whether they could potentially inform feeding behavior in current ecological contexts, is still unclear. With some exceptions, nectar amino acids are generally present at relatively low concentrations (between ∼0.1 and 15 mM in many plants, although concentrations of up to 100 mM have been observed in some species) (Nicolson 2007). These low concentrations are not likely high enough to drive preference, as even concentrations of 500 mM did not elicit a strong preference in our behavioral assay (fig. 1*e* and supplementary fig. 1*f*, Supplementary Material online). However, interestingly, in some ants, synergistic responses between certain amino acids and carbohydrates present in nectar and in caterpillar secretions have been observed (Wada et al. 2001; Blüthgen and Fiedler 2004). We wondered whether low-level amino acids might similarly potentiate hummingbird receptors, which are not homologous to insect chemosensors. Taste synergism between nucleotides and amino acids is a well-described feature of the umami receptors of great apes (Li et al. 2002; Toda et al. 2021a), but an interaction between amino acids and sugars has never been described for vertebrate T1Rs.

**Fig. 4.**
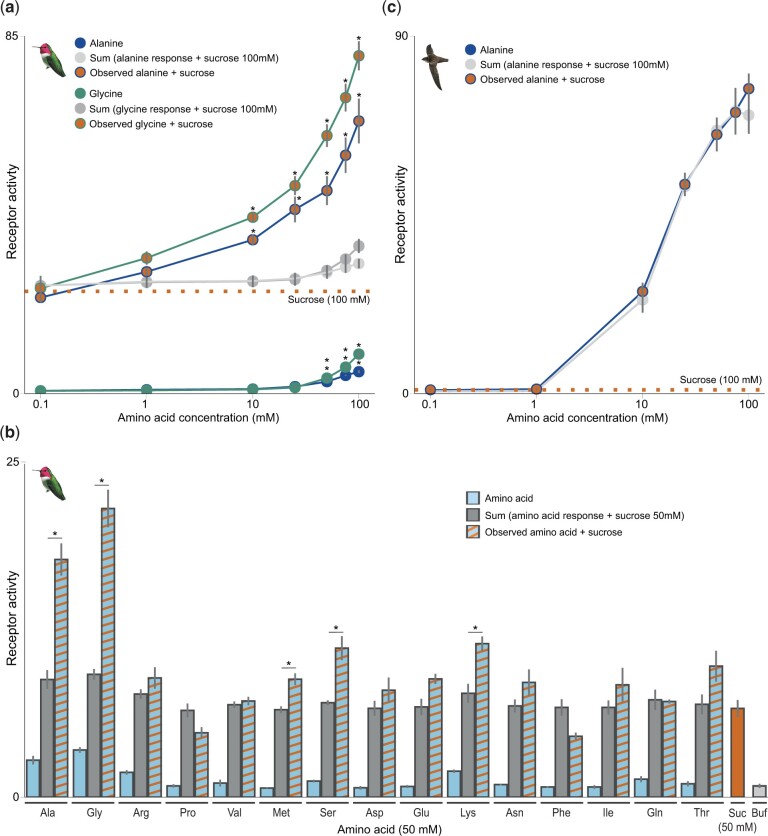
Amino acids synergistically enhance the hummingbird T1R1–T1R3 response to carbohydrates. (*a*) Dose-dependent responses of Anna’s hummingbird T1R1–T1R3 to mixes containing increasing concentrations of alanine or glycine combined with 100 mM sucrose. The observed response is higher than the combined (additive) response (gray circles, the sum of the response of 100 mM sucrose+the response of the amino acid, see Materials and Methods); notably, synergistic responses were observed at concentrations of amino acids too low to activate the receptor alone (10 and 25 mM) (*P < 0.05, two-tailed *t*-test; mean±SE, *n* = 6). The average response to 100 mM sucrose (*n* = 6) is indicated by the dashed orange line. (*b*) Synergistic responses of a panel of amino acids were assessed by comparing the response of 50 mM sucrose to combined presentations of amino acids and sucrose (50 mM each); no ligand (buf=buffer) control is also shown. The strongest synergism is observed between sucrose (50 mM) paired with alanine and glycine; responses of sucrose presented with serine, lysine, and methionine are also slightly higher than the combined response (blue, amino acid response; striped bar, amino acid+sucrose; gray, combined [additive] response; *P < 0.01, Welch’s two-tailed *t*-test; mean±SE, *n* = 4). (*c*) Synergistic responses are not observed in chimney swift T1R1–T1R3 (each concentration, *n* = 4).

To investigate whether amino acids could potentiate hummingbird taste receptors and thus enhance the taste of sugars, we examined receptor responses of Anna’s hummingbird and saw-billed hermit receptors to combinations of amino acids and sugars. Strikingly, clear synergistic responses of Anna’s hummingbird receptors were seen to some, but not all, amino acids when presented in combination with carbohydrates (fig. 4 and supplementary fig. 5, Supplementary Material online). Increasing concentrations of glycine and alanine (fig. 4*a*), but not proline (supplementary fig. 5*c*, Supplementary Material online) paired with sucrose activated the receptors in a dose-dependent manner; this response was higher than the sum of the responses of each ligand presented individually. Critically, this synergistic response is also seen at amino acid concentrations too low to activate the receptor alone (10 mM). Increased responses also occur in presentations of mixes of sucrose with other amino acids (including methionine, serine, and lysine, fig. 4*b*), as well as in combination with the other main nectar sugars (glucose and fructose, supplementary fig. 5*a* and *b*, Supplementary Material online); these responses are also exhibited by saw-billed hermit receptors (supplementary fig. 5*d* and *e*, Supplementary Material online); however, no synergy is observed in chimney swift receptors (fig. 4*c*). Many allosteric modulators have been identified for taste receptors (Zhang et al. 2008, 2010), and synergism between nucleotides and amino acids is a hallmark of the human umami taste (Nelson et al. 2002). Like the umami response of some primates, hummingbird appetitive responses are also enhanced, but in a novel way, previously undocumented in T1Rs.

## Discussion

We document diversity both in carbohydrate and in amino acid responses, and demonstrate through molecular and behavioral experiments that the hummingbird T1R1–T1R3 is a bifunctional receptor, suggesting that sweet and savory may represent the same sensory percept. By sampling multiple representatives across the radiation and profiling ancestrally reconstructed receptors, we confirmed that the acquisition of carbohydrate detection occurred prior to the diversification of extant hummingbirds, identifying a critical domain involved in sugar sensing and highlighting additional convergence on the tertiary protein level with songbird sugar detection. Moreover, we uncover a surprising synergistic response between sugars and amino acids, previously unknown in vertebrate T1Rs. These findings have broad implications for the evolution of protein function and shed new light on ecological interactions between flowering plants and a major clade of vertebrate pollinators.

The strong amino acid responses seen in both ARs (AR1 and AR2) as well as the receptors of the saw-billed hermit provide important insight into how sensory trade-offs can evolve. Whereas most tested species had higher average and total sugar responses compared with amino acid responses, the relative response index of the ancestral and hermit receptors demonstrates that hummingbird sugar detection did not initially evolve at the expense of the receptor’s ability to robustly respond to amino acids. These results suggest that the later shift in tuning is a result not of constraints at the molecular level (such as mutually exclusive changes to binding pockets) but, instead, suggests a gradual shift in how the taste system is tuned, potentially reflecting a selective advantage for increased sugar sensing relative to amino acid sensitivity. Interestingly, in songbirds, which independently evolved a distinct mechanism of T1R-based sugar sensing, responses to amino acids in some species (such as the brown-eared bulbul) are far lower than in others (such as the New Holland honeyeater, or the Atlantic canary) (Toda et al. 2021b), suggesting a convergent dynamic in which amino acid sensitivity is traded off against sugar sensitivity. Further examination of correlations between receptor responses and nectar composition—especially in hummingbirds that specialize on a single or small number of plants (such as the sword-billed hummingbird [*Ensifera ensifera*]; Abrahamczyk et al. 2014)—may clarify how plant nectar composition and hummingbird taste preferences coevolve.

Understanding the synergism between sugars and amino acids both broadens our understanding of T1R function and suggests that a hidden axis of sensory diversity may underpin certain coevolutionary interactions between plants and hummingbirds. Multiple potentiators of T1R responses have been discovered (Zhang et al. 2008, 2010) (for the mammalian sweet receptor [T1R2–T1R3], as well as for T1R1–T1R3). A hallmark of human umami taste is the potentiation of the response to glutamate (the amino acid to which human receptors are narrowly tuned) by ribonucleotides such as IMP (inosine monophosphate) (Zhang et al. 2008). Ribonucleotides are umami receptor agonists in other primates, but, concomitantly with the reduction in tuning, IMP switched from being an agonist to a modulator; this functional shift is associated with folivory across primates and may have promoted consumption of bitter or otherwise less appetitive leaves (Toda et al. 2021a). Similarly, amino acids, ancestral agonists of avian T1Rs, appear to have evolved a modulatory function in hummingbirds, enhancing responses to nectar sugars. Intriguingly, this synergistic interaction may be one reason for the unexplained presence of low-concentration amino acids in nectars: concentrations of 10 mM amino acids may be too low to be detected by hummingbirds alone but substantially enhance their responses to sugars.

As both amino acid and synergistic responses vary across hummingbird species, and as only certain amino acids act synergistically with sugars, modulating amino acid composition may be one way plants could select between different pollinators, both between hummingbird species as well as between hummingbirds and insects, similar to how some bitter South African nectars deter specific bird species but not others (Johnson et al. 2006). Low-concentration amino acids may enhance the appetitive quality of nectar sugars and help plants attract certain pollinators. Proline, for instance, a component of many nectars, does not activate or potentiate hummingbird receptors but is highly attractive to bees (Bertazzini et al. 2010). Nectar amino acids vary widely across species, in both composition and concentration (Johnson and Nicolson 2008). Variation may also exist on an intraspecific level: plants can temporally vary floral opening and nectar composition to attract different suites of pollinators (Kessler et al. 2010). By varying the composition of sugars and amino acids, plants may be able to attract specific pollinators—and may potentially even drive divergence in receptor responses of sympatric species (Muchhala et al. 2014).

Although taste receptor responses may play an important role in shaping hummingbird foraging choices, other factors can also impact hummingbird nectar preferences. Rapid postingestive responses to sugar consumption have been documented in mice (Tan et al. 2020) and may affect hummingbird nectar selection, as hinted at by studies of carbohydrate selection over longer time-scales (Reed Haisworth and Wolf 1976). Compared with nonspecialized vertebrates, hummingbirds have evolved unique physiologies to metabolize nectar sugars, including high levels of intestinal sucrase activity and glucose transport (Diamond et al. 1986; Martinez Del Rio 1990) as well as novel aspects of the sugar oxidation cascade (such as the ability to use dietary fructose as an energy source in flight muscles) (Welch and Chris 2015), which may also affect nectar choice and impact carbohydrate preferences.

## Conclusions

The substantial variation in response profiles of different hummingbird taste receptors both to carbohydrates as well as to amino acids underscores a key role for taste (Gardener and Gillman 2002) (in addition to the better-understood traits of color, [Stiles 1976] and corolla morphology, [Temeles et al. 2009]) in the coevolution between hummingbirds and plants (Dalsgaard et al. 2021). The unexpected discovery of synergistic responses broadens our understanding of how T1R taste receptors function and opens a new ecological angle for future research. Excitingly, this synergism suggests an explanation for the presence of low-concentration amino acids in many floral nectars, and may represent an unexplored way in which plants can selectively attract pollinator subsets. Profiling chemoreceptors from diverse sets of closely related species can yield valuable information about how novel protein functions evolve and can provide unexpected insight into vertebrate ecology and physiology.

## Materials and Methods

### Bird Specimens for T1R Amplification

Hummingbird tissue samples used in this study were obtained from the Museum of Comparative Zoology, Harvard University: purple-crowned fairy (*Heliothryx barotti*; MCZ 348262) and rufous-tailed hummingbird (*Amazilia tzacatl*; MCZ 335575) and the Louisiana State University Museum of Natural Science: giant hummingbird (*Patagona gigas*; B-68129), glowing puffleg (*Eriocnemis vestita*; B-31758), and greenish puffleg (*Haplophaedia aureliae*; B-33861). Samples from a black jacobin, (*Florisuga fusca*), a frilled coquette (*Lophornis magnificus*), and a saw-billed hermit (*Ramphodon naevius*) were collected in the field in Brazil (permit number: 41794-1, Genetic Heritage #A8E6064). Tissue from two edible-nest swiftlets (*Aerodramus fuciphagus*) was collected under permits obtained from Singapore (National Parks Board permit number NP_RP18-049). Anna’s hummingbird (*Calytpe anna*) and chimney swift (*Chaetura pelagica*) *T1R*s were obtained from our previous study (Baldwin et al. 2014).

### Hummingbird Natural History Data

Data on mean weight, size, elevation, and migratory status for species in this study (supplementary table 1, Supplementary Material online) are taken from Birds of the World (Billerman et al. 2020); data on range size from BirdLife (http://datazone.birdlife.org/home; last accessed November 5, 2020); and data on plants visited are from Birds of the World, as well as from images of hummingbirds visiting flowers accessioned into Macaulay Library (https://www.macaulaylibrary.org/; last accessed November 5, 2020).

### Taste Receptor Cloning and Expression

To isolate the *T1R* genes from most species, DNA was extracted from muscle tissue using the DNeasy Blood and Tissue kit (Qiagen). RNA from the black jacobin was extracted from frozen oral tissue (RNeasy Fibrous Tissue Kit, Qiagen) and was used as a template for cDNA synthesis using SMARTscribe reverse-transcriptase (Clontech). *T1R*s were amplified using primer sets initially designed from Anna’s hummingbird *T1R* sequence and modified to include information from additional hummingbird sequences as these were obtained. To identify allelic sites and to distinguish these from errors introduced during polymerase chain reactions (PCRs), multiple clones from at least three independent PCR reactions were inspected. A homology-based directional cloning approach (In-Fusion cloning, Clontech) was used to assemble deduced exons determined by comparison with Anna’s hummingbird and black jacobin transcripts; chimeric receptors were similarly constructed. Expression vectors were prepared using the pEAK10 vector as previously described (Toda et al. 2013).

### T1R Functional Assay

HEK293T cells were transfected with a calcium-dependent apophotoprotein (mt-apoclytin II), a mouse G-protein (Gα15) and an expression vector (pEAK10) encoding the cloned taste receptors (Toda et al. 2013; Baldwin et al. 2014) and plated in a 96-well plate. Cells were loaded with coelenterazine (Promega), and luminescence responses were measured using a microplate reader (Flexstation 3, Molecular Devices) immediately after solutions of a test ligand were injected into each well, as previously described (Toda et al. 2013; Baldwin et al. 2014).

Ligands were dissolved in HEPES buffer and pH-adjusted to 7.4. Unless otherwise specified (as in dose–response curves), sugars and some amino acids (alanine, serine, glycine, arginine, methionine, valine, and proline) were tested at 100 mM; all other amino acids were tested at 50 mM except leucine, which was tested at 25 mM. Abbreviations for carbohydrates are as follows: Rib, ribose; Glu, glucose; Man, mannose; Meb, melibiose; Xyl, xylose; Gal, galactose; Tre, trehalose; Raf, raffinose; Mal, maltose; Mez, melezitose; Ara, arabinose; Fru, fructose; Suc, sucrose; Scl, sucralose. Ligands were applied to transfected cells, as well as to control cells transfected only with the g-protein and with clytin, but without receptors (hereafter called untransfected); with the exception of a small response seen after application of histidine, and combinations of sugars and amino acids above 175 mM, none of the stimuli (or combinations thereof) presented here cause elevated untransfected responses. Four to six replicates (independent samples) from two to three transfections were assayed for each concentration per ligand per species. Because all the receptors of all hummingbirds examined displayed a very strong response to sucralose, to investigate the effect of possible differences in transfection efficiency across species, we divided each species’ ligand responses by the respective response to sucralose to create a normalized response (fig. 3*a* and supplementary table 2, Supplementary Material online).

Statistical analyses were performed in R 4.0.3 (R Core Team 2012). In most experiments, Welch’s one-tailed *t*-tests were used to compare responses from cells transfected with receptors against untransfected controls presented with the same ligand; *P* values were adjusted to correct for multiple hypothesis testing using the Holm method (*α* = 0.05). Because a true estimate of an additive response is difficult to calculate, to assess whether combinations of amino acids and carbohydrates synergistically affected T1R responses, the combined sum of the individual responses of each ligand was used as a proxy for an estimated additive response (calculated as the sum of the response of each ligand alone, minus the background untransfected response to no-ligand controls). The observed response was then compared with this estimated response using Welch’s two-tailed *t*-tests. For amino acid dose–response curves in figure 4 and supplementary figure 5, Supplementary Material online, significance is assessed by comparison against untransfected cells presented with no-ligand controls, using Welch’s one-tailed *t*-tests and correcting for multiple testing using the Holm method.

### Ancestral Reconstruction and Design of Chimeric Receptors

For ancestral reconstruction of taste receptors of the ancestral hummingbird (AR2) and hummingbird-swift common ancestor (AR1), we used a codon-based reconstruction using the Yang model of evolution in FastML (Ashkenazy et al. 2012), a maximum likelihood program that takes as input a multiple sequence alignment and a phylogenetic tree. Multiple sequence alignments were generated using the TranslatorX (Abascal et al. 2010) webserver and MAFFT (Katoh 2005) alignment method. Phylogenetic trees were created in Mesquite, and relationships were based on McGuire et al. (2014) and Prum et al. (2015). Sequences from hummingbirds and the chimney swift used in the ancestral reconstruction were obtained experimentally as described above or were mined from publicly available genomes using BLAST analyses (including sequences from *Alligator sinensis*, *Gallus gallus*, *Meleagris gallopavo*, *Anas platyrhyncus*, *Columba livia*, *Pseudopodoces humilis, Geospiza fortis*, *Vermivora chrysoptera*, and *Sporophila hypoxantha*, used as outgroups). All amplified hummingbird sequences were included. Marginal reconstructions from the two focal nodes were aligned to experimentally determined sequences, edited to remove spurious insertions and synthesized (GENEWIZ). Posterior probabilities of critical residues were examined: all five critical residues identified in T1R3 had a posterior probability ≥ .99, and 90% (36/40) of critical residues in the CRD-TM region of T1R1 had a posterior probability ≥0.90, with only one residue with a posterior probability less than 0.5.

To narrow down functionally relevant domains, we dissected the functional differences between the AR2 and AR1 receptor pairs using a chimeric approach (Baldwin et al. 2014; Toda et al. 2021b) focusing on the strong response to sucralose. In contrast to our previous study on hummingbirds, which specifically examined the Venus flytrap domain of T1R3 (Baldwin et al. 2014; Toda et al. 2021b), we used receptor chimeras of both T1R1 and T1R3 and investigated the role of different domains. To narrow down residues within a functionally relevant section of the VFT, we next explored the use of three criteria, and tested a multiple point-mutant chimera that contained residues that differed between ancestral pairs that were a) radical changes (following for instance Lee et al. [2018]), b) had a BLOSUM62 (Henikoff and Henikoff 1992) score of 0 or below (to examine amino acid substitutions that occur relatively infrequently), and c) were evolutionarily conserved between AR2 and all extant hummingbird sequences. To examine the role of radical changes or amino acids with low BLOSUM62 scores in T1R1, we synthesized TMDs containing residue subsets and generated receptor chimeras as described above.

### Tests of Positive Selection

For analyses of positive selection, alignments of *T1R1* and *T1R3* were created as described above, including a larger (and nonoverlapping) set of outgroup sequences. All outgroups were obtained (and manually curated) from publicly available genomes, apart from the edible-nest swiftlet sequences, which were mined from unpublished draft assemblies and transcriptomes generated for another project; tree topology (supplementary fig. 3*a*, Supplementary Material online) was specified following McGuire et al. (2014) and Prum et al. (2015). Branch and site models were tested using CODEML in the PAML v4.8 (Yang 2007) package, as well as with aBSREL (Smith et al. 2015) and MEME (Murrell et al. 2012) from the Hyphy package. Branches showing signatures of positive selection in aBSREL (uncorrected *P* < 0.05) are indicated in figure 2*a*; branch-site tests (Zhang et al. 2005) on the branch leading to the ancestor of all hummingbirds suggested positive selection in *T1R3*, but not *T1R1*; likelihood ratio tests and χ^2^ tests of significance were used to compare model likelihoods. Per-branch ratios of nonsynonymous to synonymous rates (ω) are taken from free-ratio models (supplementary fig. 3*b*, Supplementary Material online). Site models (M1a vs. M2, M7 vs. M8) were compared using likelihood ratio tests; site-wise ω and sites predicted to be under selection by Bayes empirical Bayes (Yang 2005) analyses from M8, as well as sites determined to be positively selected by MEME analyses (*P* < 0.05), are displayed in supplementary figure 3*c*, Supplementary Material online.

### Principal Component Analysis

Phylogenetic principal components analysis (phyloPCA) was performed in R 4.0.3 using the phyl.pca() function in the phytools package (Revell 2012) on both raw response data (fig. 3*c*) as well as on log-transformed responses (supplementary fig. 4*b*, Supplementary Material online) (including the chimney swift). Tree topology was specified following McGuire et al. (McGuire et al. 2014). For branch lengths, branch-specific mean d*S* (synonymous changes per synonymous site) values were calculated using the free ratio model of the CODEML package in PAML.

### Relative Response Index

To assess when changes in the overall response to amino acids relative to carbohydrates occurred in the hummingbird phylogeny, we first calculated a response index (sum sugar responses−sum amino acid responses)/(sum sugar responses+sum amino acid responses). This index (from −1 to 1) is mapped on the hummingbird phylogeny in figure 3*b* using the contmap() function in the package phytools (Revell 2012) (branch lengths shown reflect values from the d*S* tree calculated by PAML free-ratio models).

### Examination of Adaptive Shifts Using Reversible-Jump Models

Next, we fit a Bayesian reversible jump Ornstein-Uhlenbeck model to the average amino acid or sugar values for each species, as well as the difference between the averages, to test whether there was support for shifts in the optimal ligand response across the evolutionary history of the tested species. We used the package bayou (Uyeda and Harmon 2014) in R 4.0.3. We began by making the d*S* tree ultrametric using penalized likelihood. We then used the make.prior() function of bayou to set priors on parameters; in particular, we used a Poisson distribution with a lambda of 1 on the expected number of shifts in response optima. We ran two MCMC chains for 2 million generations each, sampling every 100 generations. After discarding the first 30% of retained samples as burn-in, we compared Gelman’s R for each parameter to confirm convergence of the chains. Since Gelman’s R was below 1.1 for all parameters (Uyeda and Harmon 2014), we combined the results into a single set of samples. However, the posterior probabilities of most shifts were low (posterior probability less than 0.1). Our inability to confidently detect well-supported shift configurations with this approach is likely due to our relatively limited taxon sampling; expanded taxon sets may enable determination of other shifts within Apodiformes.

### Homology Model

The homology model of the heterodimer of AR2 T1R1 and T1R3 was constructed as previously described (Baldwin et al. 2014; Toda et al. 2021b) using the program Maestro (Schrödinger Suite 2019-1) with the active form of mGluR5 (PDB ID: 6N51) used as a template. Visualization of highlighted residues was performed in Discovery Studio Visualizer (Dassault Systèmes).

### Behavioral Assays

Brief-access behavioral tests were approved by the Institutional Animal Care and Use Committee at Harvard University and were conducted with wild hummingbirds at a field site in the Santa Monica Mountains, as previously described (Baldwin et al. 2014). Anna’s hummingbirds (*Calypte anna*) were the main species present, and black-chinned hummingbirds (*Archilochus alexandri*) also frequently visited the feeders; occasional visits from Allen’s hummingbirds (*Selasphorus sasin*) were also documented. Adult male hummingbirds of different species were clearly distinguishable, but females (and juveniles) could not always be reliably told apart in all trials; drinking bout lengths are therefore reported both for Anna’s hummingbird males as well as for the entire population of all individuals (all three species). Similar results were observed between species and sexes in comparisons with sucrose, glucose, and fructose, and between Anna’s hummingbird males and the entire population for amino acid tests (fig. 1 and supplementary fig. 1, Supplementary Material online).

During testing, a circular feeder array consisting of 6 disposable tubes containing stimuli (4 ml) and covered with wire-secured flower caps from commercial hummingbird feeders was filmed at 60 frames per second. Feeders contained duplicate presentations of either A) 500 mM sucrose, fructose, and glucose or B) 500 mM fructose, 500 mM glucose and water, or presentations in triplicate C) of water and an amino acid. Although low concentrations (500 mM) of some amino acids elicited a few long drinking bouts, these did not appear to be strongly preferred in our assay; therefore, 1.5 M concentrations were tested for alanine, glycine, serine, and proline. Trials lasted between 10 and 20 min; sucrose (500 mM) was presented in all feeder positions between trials. As individuals were not color banded, each visit was treated as a sample. Amino acid trials were repeated over multiple field seasons to obtain sufficient sampling by adult Anna’s hummingbird males; similar results were observed in each season and data were pooled across years.

Trials were scored by an observer blinded to the relative position of stimuli. Drinking bouts were measured as the time between the entry of the tip of the bill (or tongue, if visible) into the feeder, and the initiation of withdrawal behavior. Birds sampled often from all feeders in the array, with longer bouts (∼1–2 s) observed less frequently; differences between bout length distributions between different pairs of stimuli or between stimuli and water controls were assessed using Kolmogorov–Smirnov tests (fig. 1 and supplementary fig. 1, Supplementary Material online) performed in R 4.0.3.

## Supplementary Material

Supplementary data are available at *Molecular Biology and Evolution* online.

## Supplementary Material

msab367_Supplementary_Data

## Data Availability

The data underlying this article are available in the Dryad Digital Repository, at https://doi.org/10.5061/dryad.v6wwpzgxk; sequences used for functional testing are accessioned in GenBank (accession numbers OM142608-OM142621).
