# Supplementary material for: Synergism, Bifunctionality, and the Evolution of a Gradual Sensory Trade-off in Hummingbird Taste Receptors
Source: Mol Biol Evol. 2022 Jan 3;39(2):msab367. doi: 10.1093/molbev/msab367 (PMC8826506; doi:10.1093/molbev/msab367)
Supplement: msab367_Supplementary_Data [file msab367_supplementary_data.pdf]

Supplemental information for "Synergism, bifunctionality, and the evolution of a gradual sensory trade-off in hummingbird taste receptors"

Glenn Cockburn, Meng-Ching Ko, Keren R. Sadanandan, Eliot T. Miller, Tomoya Nakagita, Amanda Monte, Sungbo Cho, Eugeni Roura, Yasuka Toda, Maude W. Baldwin*

***** corresponding author: mbaldwin@orn.mpg.de

Supplemental Figure 1. Behavioral responses of wild hummingbirds to different combinations of sugars and amino acids.

Supplemental Figure 2. Chimeric dissection of ancestral pairs (AR1 and AR2).

Supplemental Figure 3. Tests of positive selection in the hummingbird clade.

Supplemental Figure 4. Responses of hummingbird T1R1-T1R3 to carbohydrates and amino acids.

Supplemental Figure 5. Synergistic responses of Anna's hummingbird and saw-billed hermit T1R1-T1R3 receptors to combinations of amino acids and sugars.

Supplemental Table 1. Natural history traits of tested hummingbirds.

Supplemental Table 2. Normalized ligand responses.

Supplemental Table 3. Location of AR2 residues in T1R1 CRD-TMD**.**

Supplemental Table 4: Sample sizes for behavioral trials with wild hummingbirds.

Supplemental Figure 1. Behavioral responses of wild hummingbirds to different combinations of sugars and amino acids.

(a-f) Drinking bout lengths from a wild population of hummingbirds (primarily Anna's and black-chinned hummingbirds) presented with sugars and amino acids. **(a-c)** Responses to simultaneous presentation of 500 mM carbohydrates for **(a)** all birds (all species and sexes), as well as responses separated by species and sex: **(b)** black-chinned males (see Fig. 1c for Anna's males), and **(c)** females (all species). All groups show strong sucrose preferences. **(d)** In the absence of sucrose, fructose (500 mM) is preferred, data from both sexes and all species combined. **(e)** Responses from all birds for trials with amino acid solutions (1.5 M). Tests with three receptor agonists—alanine, serine, and glycine—but not proline (which does not activate the hummingbird receptor, Fig. 1b) elicited longer drinking bouts than paired water controls (see Fig. 1d for Anna's hummingbird males). **(f)** Lower-concentration (500 mM) solutions of serine evoked some long bouts, but the difference between bout lengths compared to water controls was not significant (see Fig. 1e for Anna's hummingbird males). Asterisks denote $p < 0.05$, two-sample Kolmogorov-Smirnov tests; see Supplemental Table 4 for sample sizes.

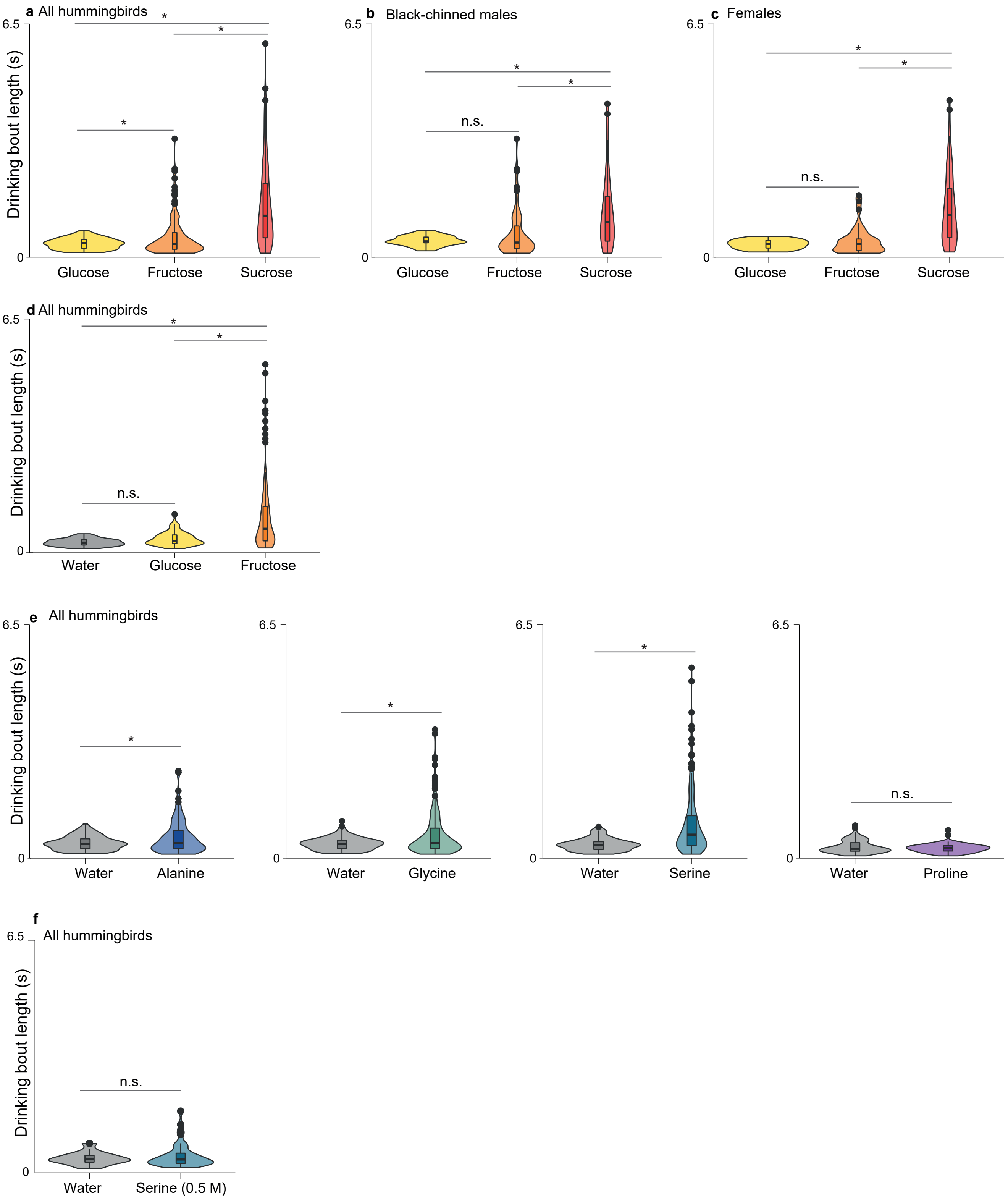

Supplemental Figure 1

Supplemental Figure 2. Chimeric dissection of ancestral pairs (AR1 and AR2).

(**a**) Schematic of T1R1-T1R3 heterodimer indicating receptor domains, and diagram depicting the sequence identities (as a percentage of sequence length, number of coding differences given in parentheses) between receptors from different species (chicken, chimney swift, black jacobin, Anna's hummingbird) and ancestral nodes (AR1 and AR2). (**b**) Alignment of 19 previously-identified residues (Baldwin et al. 2014) with our ancestral receptors and the hummingbirds cloned in this study; 10 of these 19 residues differ between ancestors, and 9 (highlighted in yellow) are conserved across the 8 examined hummingbirds. **(c)** Responses of ancestral receptors to sucrose, sucralose, alanine and no-ligand control. AR1 responds only to amino acids, whereas AR2 responds robustly both to sugars as well as amino acids ($n = 6$, mean ± SE; $*p < 0.05$). Mixed pairs of heterodimers comprising AR1 & AR2 fail to respond strongly to sucrose and sucralose. (**d**) Domain chimeras between AR1 and AR2 demonstrate a critical role of T1R1 CRD+TMD and T1R3 VFT. (**e**) The domain combination underlying songbird sweet taste acquisition (T1R1-VFT and T1R3-CRD-TMD (Toda et al. 2021)) does not evoke a response to sugars or sweeteners in hummingbirds**.** In hummingbirds, the strongest sucralose responses were observed when both domains were present. (**f**) Dissection of the T1R3 VFT domain reveals highest responses from the second domain quarter, consistent with previous findings (Baldwin et al. 2014). (**g**) When expressed with AR1 T1R1, this second VFT domain quarter does not show any sugar or sweetener response, underscoring the role of the CRD-TM domain of T1R1. (**h**) By examining patterns of conservation and radical change (see Methods), a subset of 5 residues from the second quarter of the VFT were tested with the AR2 CRD-TMD chimera; interestingly, responses to sucralose from this smaller residue subset were elevated compared with the responses from the second domain chimera (when both were co-expressed with the CRD-TMD chimera) in f. (**i**) A similar approach to narrow down CRD-TMD residues, from an initial 40 differences between receptor pairs, to 22 (using radical changes) or 17 (using BLOSUM62 similarity scores) did not capture the relevant residues, and no response to sucralose was recovered. (**j**) Alignment of a subset of the dissected region of the T1R3 domain, showing the five examined residues (yellow highlighting; asterisks indicate residues discussed in Baldwin et al. (2014)). A more extensive chimeric dissection will be required to uncover the full set of necessary and sufficient residues across both T1R1 and T1R3 underlying hummingbird sugar sensing.

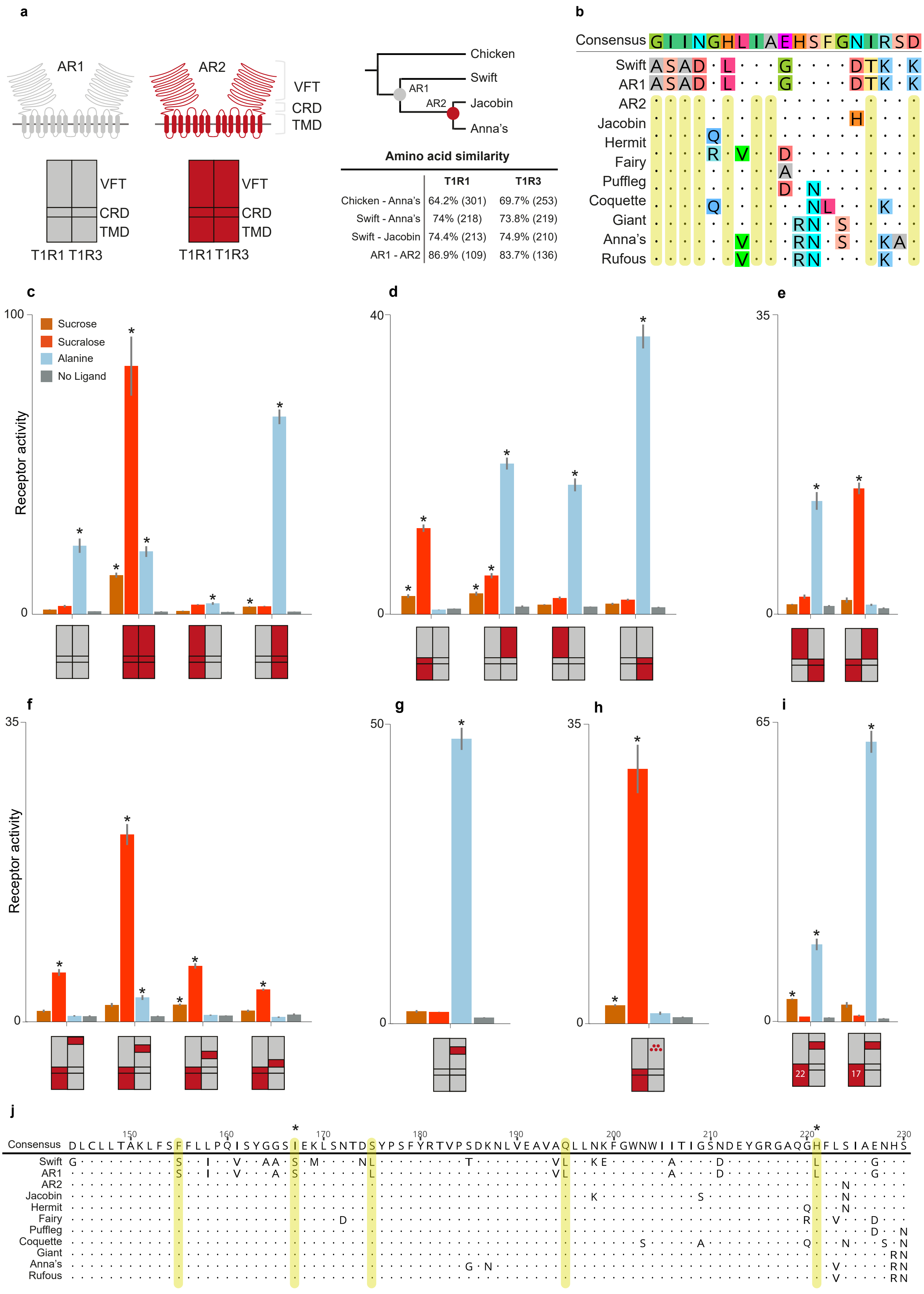

Supplemental Figure 2

Supplemental Figure 3. Tests of positive selection in the hummingbird clade. **(a)** Tests of positive selection (aBSREL branch models (Smith et al. 2015)) suggest selection on T1R3 at the base of the hummingbird radiation (red branches: uncorrected *p*-value < 0.05). (**b**) In free-ratio models (CODEML; PAML v4.8a, (Yang 2007)), T1R3 has higher ratios of non-synonymous to synonymous rates (ω) on many branches compared with T1R1. **(c)** Site-wise ω values from M8 (CODEML) are shown, as well as the location of residues predicted to be under selection by site models implemented in CODEML (orange, BEB sites from M8 (Yang 2005); asterisks indicate Pr > 0.95) or in MEME (Murrell et al. 2012) (yellow; $p < 0.05$). Little overlap is seen with sites determined by chimeric dissection (black) with the exception of site 175 (T1R3), and five sites in T1R1 (see Supplemental Table 3).

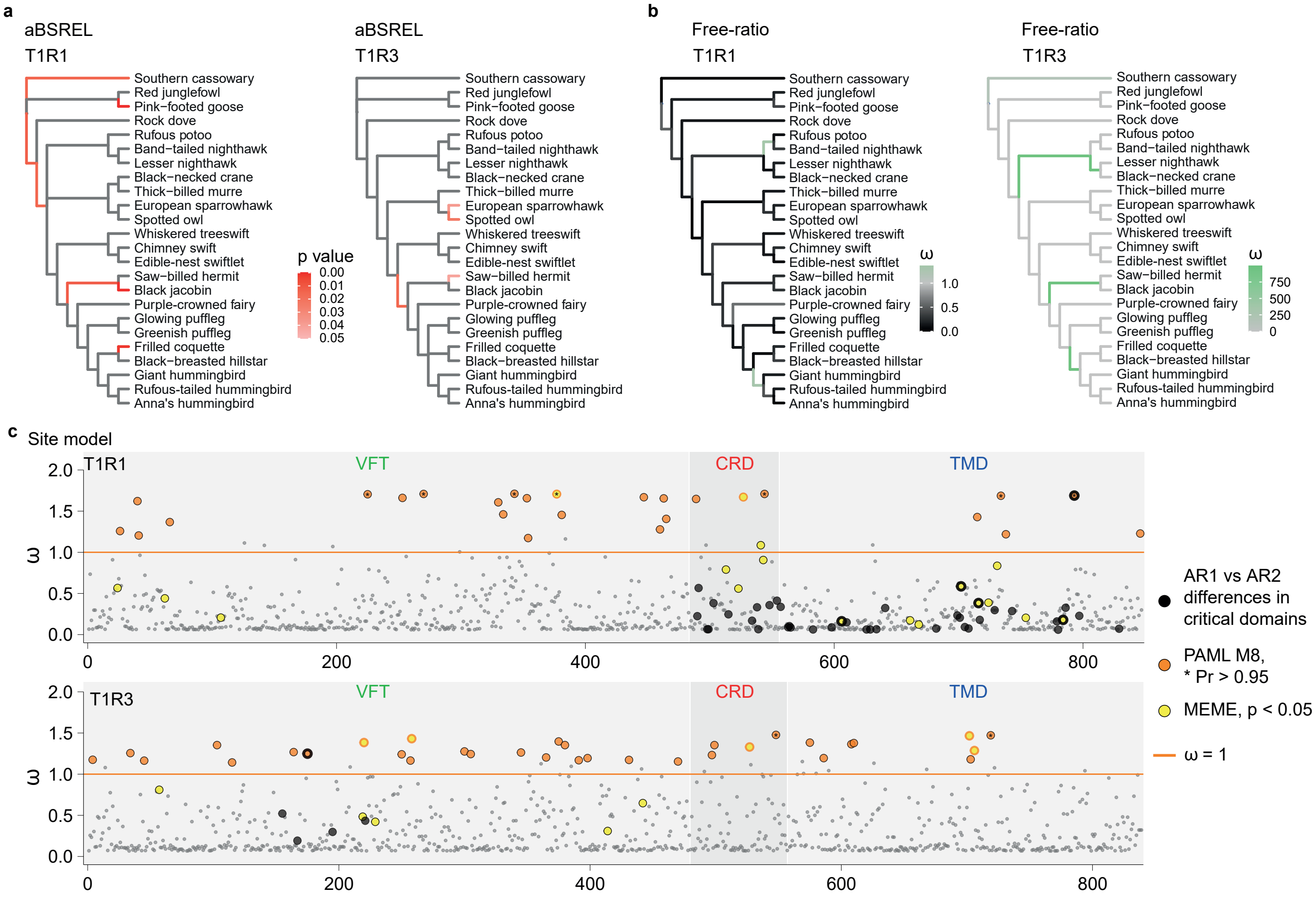

Supplemental Figure 3

Supplemental Figure 4. Responses of hummingbird T1R1-T1R3 to carbohydrates and amino acids.

(a) Bar plots of single ligand plots (n = 6, mean ± SE) for eight hummingbirds, the swift and the two ancestral reconstructions as well as responses from untransfected control cells. Carbohydrates and sucralose are in light orange, amino acids are in light blue and no-ligand controls are shown in gray. Asterisks denote p-values < 0.05 after multiple correction by ligand using the Holm method. **(b)** Phylogenetic PCA (phyloPCA) of log-transformed responses including the swift. **(c)** Hummingbird-wide average; responses are similar to Figure 3a (see also Supplemental Table 2).

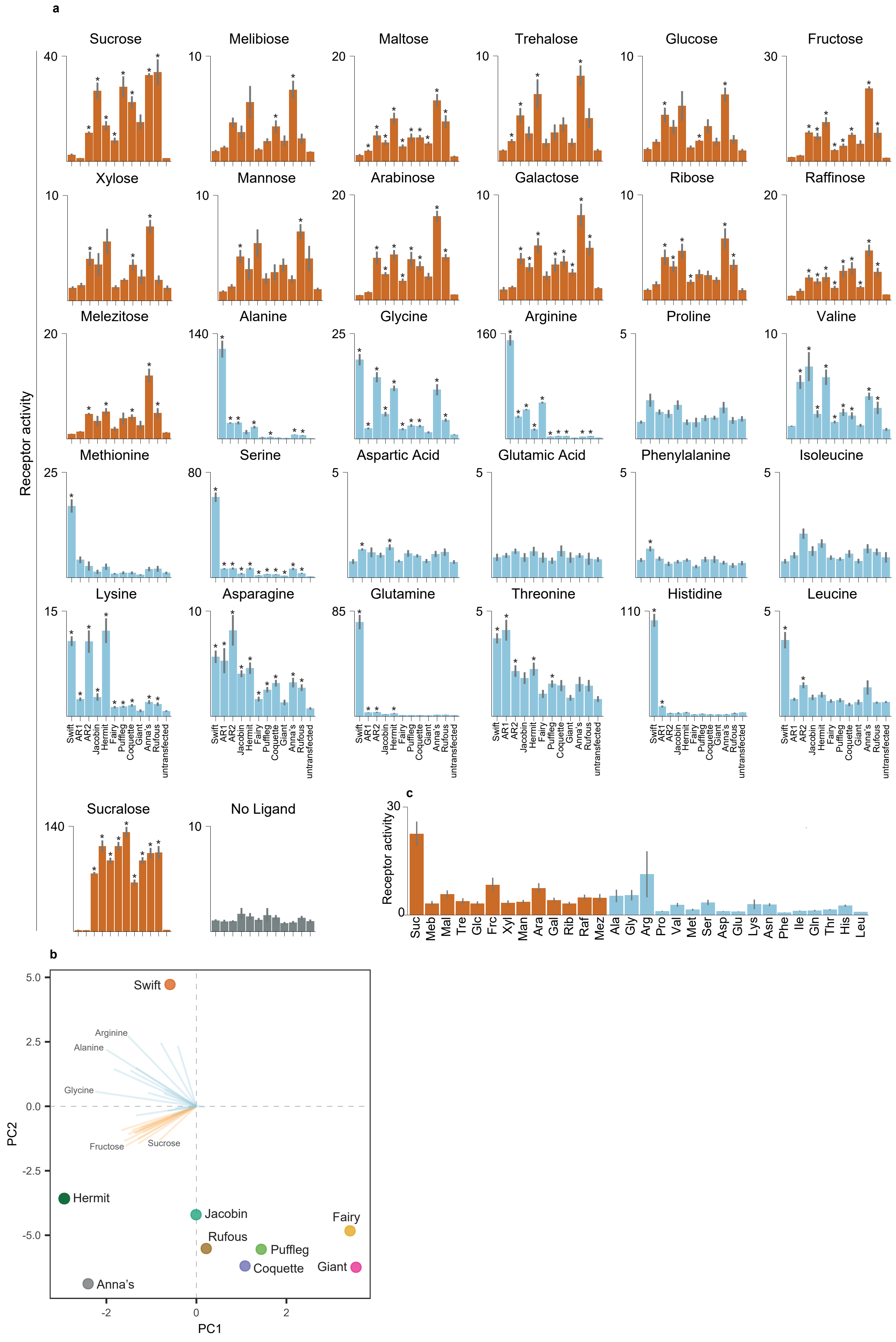

Supplemental Figure 4

Supplemental Figure 5. Synergistic responses of Anna's hummingbird and saw-billed hermit T1R1-T1R3 receptors to combinations of amino acids and sugars.

(a,b) Alanine also enhances responses to two monosaccharides (fructose and glucose) commonly found in nectar; observed responses larger than simple additive responses are seen when both sugars are presented in combination with 10 mM alanine (*$p < 0.05$, Welch's two-tailed *t*-test; mean ± SE, n = 4). The average values for 100 mM fructose and glucose (n = 6 for each) are indicated by dashed orange and yellow lines. **(c)** Proline does not enhance responses of the Anna's T1R1-T1R3 receptor when combined with sucrose (*$p < 0.05$, Welch's two-tailed *t*-test; mean ± SE, n = 4; average value for 100 mM sucrose (n = 6) indicated by dashed line). **(d)** Synergy between glycine and sucrose is also observed in responses of the saw-billed hermit, from concentrations of 25 mM onward (*$p < 0.05$, Welch's two-tailed *t*-test; mean ± SE, n = 4; average value for 100 mM sucrose (n = 6) indicated by dashed line). **(e)** Saw-billed hermit receptors show synergism between 50 mM sucrose and 7 of 15 tested amino acids (blue, amino acid response; striped bar, amino acid + sucrose; gray, estimated additive response; *$p < 0.01$, Welch's two-tailed *t*-test; mean ± SE, n = 4); no ligand controls ('buf' = buffer) are shown.

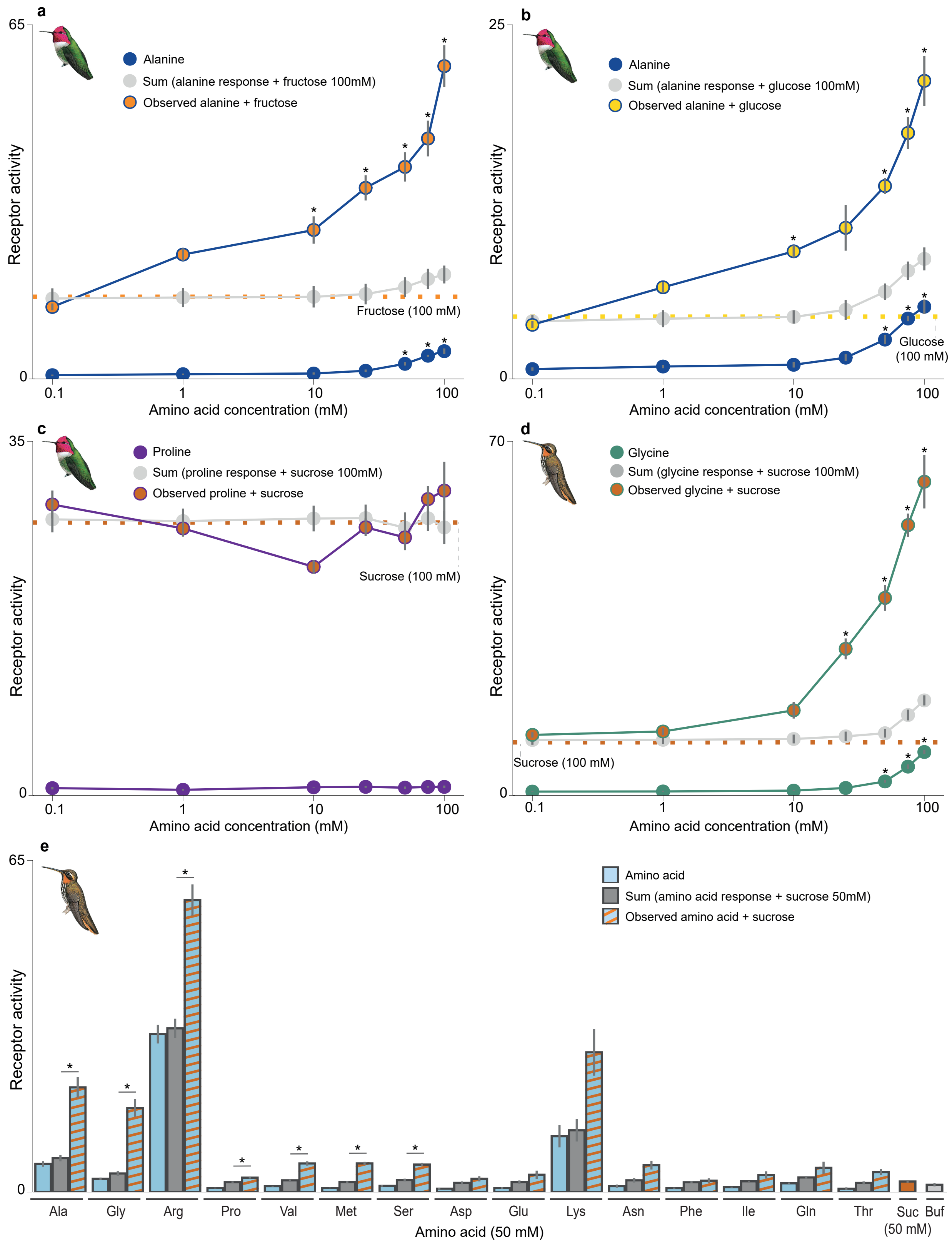

Supplemental Figure 5

Supplemental Table 1. Natural history traits of tested hummingbirds. Data on the mean weight (g), size (cm), elevation (m), migratory status, range size (km^2) and plant families visited (see Methods) are shown for the eight species of hummingbird tested in this study.

Supplemental Table 1. Natural history traits of tested hummingbirds

Species	Mean weight (g)	Mean size (cm)	Mean elevation (m)	Migratory	Range size (km^2)	Plant families visited (Birds of the World)	Plants visited (Macaulay library)
Black jacobin *Florisuga fusca*	8	12.5	456.4	Yes (primarily within Brazil)	1,790,000	Euphorbiaceae, Malvaceae, Myrtaceae, Fabaceae, Bromeliaceae	Variety of trees, shrubs, and epiphytes including numerous Fabaceae and Bignoniaceae
Saw-billed hermit *Ramphodon naevius*	8.2	15	515.5	No	517,000	Bromeliaceae,Costaceae, Fabaceae, Gesneriaceae, Heliconiaceae, Lobeliaceae, Rubiaceae, Malvaceae, Marcgraviaceae, Caricaceae	Shrubs and epiphytes, particularly *Heliconia*, *Costus*, *Justicia*, *Centropogon*, etc.
Purple crowned fairy *Heliothryx barroti*	5.47	11	373.9	No	2,200,000	Passifloraceae, Bromeliaceae, Heliconiaceae	Wide variety of plants, but frequently seen at Fabaceae trees
Greenish puffleg *Haplophaedia aureliae*	5.5	10	1961.7	No	435,000		Small flowering plants, particularly Ericaceae
Frilled coquette *Lophornis magnificus*	2.66	7.5	561.5	No	3,080,000	Verbenaceae, Ranunculaceae, Rubiaceae, Malvaceae, Leguminosae, Myrtaceae, Acanthaceae, Polemoniaceae, Lamiaceae, Bromeliaceae	Small flowering plants, e.g. *Lantana*, *Stachytarpheta*, etc.
Giant hummingbird *Patagona gigas*	19	21	3066.0	No	3,690,000	Scrophulariaceae,Passifloraceae, Loranthaceae,Myrtaceae, Mutiseae, Solanaceae, Campanulaceae, Bromeliaceae	*Puya* (and introduced *Agave*), Cactaceae, variety of other shrubs
Anna's hummingbird *Calypte anna*	4.5	10	640.6	Yes	1,580,000	Many plant families visited including non-native. Ericaceae, Phyrmaceae, Lamiaceae, Plantaginaceae, Onagraceae,	Wide variety, particularly small herbs in the order Lamiales

						Ranunculaceae, Thymelaeaceae	
Rufous-tailed hummingbird *Amazilia tzacatl*	5	9	404.4	No	3,770,000	Heliconiaceae, Musaceae, Rubiaceae	Wide variety of trees and shrubs

Supplemental Table 2. Normalized ligand responses.

All eight hummingbirds responded most strongly to sucralose (Supplemental Figure 4a); therefore, to account for possible differences in transfection efficiency, we divided each species' ligand responses by the respective response to sucralose (Fig. 3a). Relative average responses were consistent in both normalized and non-normalized comparisons. Sucrose elicited the strongest response of all carbohydrates, followed by fructose and arabinose. Across species, sucrose responses were always the highest, and most species responded more to fructose than to arabinose. For amino acids, the hummingbird-wide average response to arginine was higher than to other amino acids in both comparisons (and arginine responses were highest for most species), followed by glycine and alanine. In Anna's hummingbirds, glycine evoked the highest amino acid response.

Supplemental Table 2. Normalized ligand response

Species	Mean amino acid / mean sucralose																
	Ala	Gly	Arg	Pro	Val	Met	Ser	Asp	Glu	Phe	Ile	Lys	Asn	Gln	Thr	His	Leu
Jacobin	0.08	0.05	0.12	0.01	0.02	0.01	0.03	0.01	0.01	0.01	0.01	0.02	0.04	0.01	0.02	0.03	0.01
Hermit	0.17	0.13	0.58	0.02	0.06	0.03	0.07	0.02	0.01	0.01	0.02	0.13	0.05	0.03	0.02	0.04	0.01
Fairy	0.02	0.02	0.03	0.01	0.01	0.01	0.02	0.01	0.01	0.01	0.01	0.01	0.01	0.01	0.01	0.02	0.01
Puffleg	0.02	0.02	0.03	0.01	0.02	0.01	0.02	0.01	0.01	0.00	0.01	0.01	0.02	0.01	0.01	0.02	0.01
Coquette	0.02	0.05	0.07	0.02	0.03	0.02	0.04	0.02	0.02	0.01	0.02	0.02	0.05	0.01	0.02	0.03	0.01
Giant	0.01	0.02	0.02	0.01	0.01	0.01	0.01	0.01	0.01	0.01	0.01	0.01	0.01	0.01	0.01	0.02	0.01
Anna's	0.06	0.11	0.03	0.01	0.04	0.02	0.06	0.01	0.01	0.01	0.01	0.02	0.03	0.01	0.01	0.02	0.01
Rufous	0.04	0.04	0.04	0.01	0.03	0.02	0.03	0.01	0.01	0.01	0.01	0.02	0.03	0.01	0.01	0.03	0.01
Grand Mean	0.05	0.05	0.12	0.01	0.03	0.01	0.04	0.01	0.01	0.01	0.01	0.03	0.03	0.01	0.02	0.03	0.01
SE	0.02	0.01	0.06	0.00	0.01	0.00	0.01	0.00	0.00	0.00	0.00	0.01	0.00	0.00	0.00	0.00	0.00

Species	Mean sugar / mean sucralose												
	Suc	Meb	Mal	Tre	Glc	Frc	Xyl	Man	Ara	Gal	Rib	Raf	Mez
Jacobin	0.24	0.02	0.03	0.02	0.03	0.06	0.03	0.03	0.04	0.03	0.03	0.03	0.03
Hermit	0.14	0.06	0.09	0.07	0.06	0.12	0.06	0.06	0.09	0.05	0.05	0.05	0.05
Fairy	0.07	0.01	0.02	0.02	0.01	0.03	0.01	0.02	0.03	0.02	0.02	0.02	0.02
Puffleg	0.21	0.01	0.03	0.02	0.01	0.03	0.01	0.02	0.06	0.03	0.02	0.04	0.03
Coquette	0.34	0.05	0.07	0.05	0.05	0.12	0.05	0.05	0.10	0.06	0.04	0.09	0.06
Giant	0.16	0.02	0.04	0.02	0.02	0.05	0.02	0.02	0.05	0.03	0.02	0.03	0.03
Anna's	0.31	0.07	0.11	0.08	0.06	0.20	0.07	0.06	0.15	0.08	0.06	0.09	0.12
Rufous	0.32	0.02	0.07	0.04	0.02	0.08	0.02	0.04	0.08	0.05	0.03	0.05	0.05
Grand Mean	0.23	0.03	0.06	0.04	0.03	0.09	0.03	0.04	0.08	0.04	0.03	0.05	0.05
SE	0.03	0.01	0.01	0.01	0.01	0.02	0.01	0.01	0.01	0.01	0.00	0.01	0.01

Supplemental Table 3. Location of AR2 residues in T1R1 CRD-TMD.

Number of differences (between AR1 and AR2) for each transmembrane helix (TM), extracellular loop (ECL), intracellular loop (ICL) and C-terminus are presented separately. Most differences (standardized by region length) occur in ECL2 and TM7. Predicted selected sites from MEME and CODEML analyses are indicated, as are residues that overlap with sites of known function from other studies.

Supplemental Table 3. Location of AR2 residues in T1R1 CRD-TMD

Domain	Length	Synonymous sites	Non-synonymous sites	Proportion non-synonymous sites	MEME & PAML sites	Known functional sites
CRD	68	57	11	0.16		#491 sugars (Toda et al. 2021) #548 thaumatin (Masuda et al. 2013)
TM1	24	20	4	0.17		
ICL1	13	12	1	0.08		
TM2	21	18	3	0.14	#606 MEME	
ECL1	5	5	0	0.00		
TM3	34	30	4	0.12		#626 sugars (Toda et al. 2021) and cyclamate (Jiang et al. 2005), #641 sugars (Toda et al. 2021)
ICL2	14	14	0	0.00		
TM4	26	25	1	0.04		
ECL2	19	14	5	0.26	#702 MEME	
TM5	29	26	3	0.10	#716 MEME	
ICL3	2	2	0	0.00		
TM6	28	27	1	0.04		#743 monosodium glutamate (Dang et al. 2019)
ECL3	7	7	0	0.00		
TM7	26	20	6	0.23	#784 MEME #793 PAML(M8)	#784 lactisol (Xu et al. 2004), #786 methional (Toda et al. 2018)
C-terminus	25	24	1	0.04		
SUM	341	301	40	0.12		

Supplemental Table 4: Sample sizes for behavioral trials with wild hummingbirds. Top: number of visits (all species and both sexes, pooled and shown separately) to feeders containing carbohydrates or water. Occasional visits by Allen's hummingbirds are included in the "all-birds" column. Bottom: number of visits (all birds, and Anna's hummingbird males shown separately) to feeders containing water or amino acids (trials were repeated 2 – 4 times and visits were pooled).

Supplemental Table 4. Sample sizes for behavioral trials with wild hummingbirds

Carbohydrates				
	All birds	Anna's males	Black-chinned males	Females
Sucrose	138	24	28	78
Fructose	144	45	63	34
Glucose	59	14	16	28
	All birds			
Fructose	164			
Glucose	59			
Water	57			
**Amino acids**				
	All birds		Anna's males	
	Amino acid	Water	Amino acid	Water
Alanine (1.5M)	98	114	34	50
Glycine (1.5M)	223	110	42	31
Serine (1.5M)	185	79	65	26
Proline (1.5M)	62	74	25	32
	All birds		Anna's males	
Serine (500 mM)	36	28	22	14

References

Baldwin MW, Toda Y, Nakagita T, O'Connell MJ, Klasing KC, Misaka T, Edwards S V., Liberles SD. 2014. Evolution of sweet taste perception in hummingbirds by transformation of the ancestral umami receptor. Science (80-). 345(6199):929–933. doi:10.1126/science.1255097. http://www.sciencemag.org/cgi/doi/10.1126/science.1255097.

Dang Y, Hao L, Cao J, Sun Y, Zeng X, Wu Z, Pan D. 2019. Molecular docking and simulation of the synergistic effect between umami peptides, monosodium glutamate and taste receptor T1R1/T1R3. Food Chem. 271(March 2018):697–706. doi:10.1016/j.foodchem.2018.08.001. https://doi.org/10.1016/j.foodchem.2018.08.001.

Jiang P, Cui M, Zhao B, Snyder LA, Benard LMJ, Osman R, Max M, Margolskee RF. 2005. Identification of the cyclamate interaction site within the transmembrane domain of the human sweet taste receptor subunit T1R3. J Biol Chem. 280(40):34296–34305. doi:10.1074/jbc.M505255200.

Masuda T, Taguchi W, Sano A, Ohta K, Kitabatake N, Tani F. 2013. Five amino acid residues in cysteine-rich domain of human T1R3 were involved in the response for sweet-tasting protein, thaumatin. Biochimie. 95(7):1502–1505. doi:10.1016/j.biochi.2013.01.010. http://dx.doi.org/10.1016/j.biochi.2013.01.010.

Murrell B, Wertheim JO, Moola S, Weighill T, Scheffler K, Kosakovsky Pond SL. 2012. Detecting individual sites subject to episodic diversifying selection. Malik HS, editor. PLoS Genet. 8(7). doi:10.1371/journal.pgen.1002764. https://dx.plos.org/10.1371/journal.pgen.1002764.

Smith MD, Wertheim JO, Weaver S, Murrell B, Scheffler K, Kosakovsky Pond SL. 2015. Less is more: an adaptive branch-site random effects model for efficient detection of episodic diversifying selection. Mol Biol Evol. 32(5):1342–1353. doi:10.1093/molbev/msv022. https://academic.oup.com/mbe/article-lookup/doi/10.1093/molbev/msv022.

Toda Y, Ko M, Liang Q, Miller ET, Rico-Guevara A, Nakagita T, Sakakibara A, Uemura K, Sackton T, Hayakawa T, et al. 2021. Early origin of sweet perception in the songbird radiation. Science (80-). 373(6551):226–231. doi:10.1126/science.abf6505. https://www.sciencemag.org/lookup/doi/10.1126/science.abf6505.

Toda Y, Nakagita T, Hirokawa T, Yamashita Y, Nakajima A, Narukawa M, Ishimaru Y, Uchida R, Misaka T. 2018. Positive/negative allosteric modulation switching in an umami taste receptor (T1R1/T1R3) by a natural flavor compound, methional. Sci Rep. 8(1):1–13. doi:10.1038/s41598-018-30315-x. http://dx.doi.org/10.1038/s41598-018-30315-x.

Xu H, Staszewski L, Tang H, Adler E, Zoller M, Li X. 2004. Different functional roles of T1R subunits in the heteromeric taste receptors. Proc Natl Acad Sci. 101(39):14258–14263. doi:10.1073/pnas.0404384101. http://www.pnas.org/cgi/doi/10.1073/pnas.0404384101.

Yang Z. 2005. Bayes empirical bayes inference of amino acid sites under positive selection. Mol Biol Evol. 22(4):1107–1118. doi:10.1093/molbev/msi097. https://academic.oup.com/mbe/article-lookup/doi/10.1093/molbev/msi097.

Yang Z. 2007. PAML: a program package for phylogenetic analysis by maximum likelihood 4.8a. Mol Biol Evol. 24(5):1586–1591.
